# Genetic Structure of Europeans: A View from the North–East

**DOI:** 10.1371/journal.pone.0005472

**Published:** 2009-05-08

**Authors:** Mari Nelis, Tõnu Esko, Reedik Mägi, Fritz Zimprich, Alexander Zimprich, Draga Toncheva, Sena Karachanak, Tereza Piskáčková, Ivan Balaščák, Leena Peltonen, Eveliina Jakkula, Karola Rehnström, Mark Lathrop, Simon Heath, Pilar Galan, Stefan Schreiber, Thomas Meitinger, Arne Pfeufer, H-Erich Wichmann, Béla Melegh, Noémi Polgár, Daniela Toniolo, Paolo Gasparini, Pio D'Adamo, Janis Klovins, Liene Nikitina-Zake, Vaidutis Kučinskas, Jūratė Kasnauskienė, Jan Lubinski, Tadeusz Debniak, Svetlana Limborska, Andrey Khrunin, Xavier Estivill, Raquel Rabionet, Sara Marsal, Antonio Julià, Stylianos E. Antonarakis, Samuel Deutsch, Christelle Borel, Homa Attar, Maryline Gagnebin, Milan Macek, Michael Krawczak, Maido Remm, Andres Metspalu

**Affiliations:** 1 Institute of Molecular and Cell Biology, University of Tartu, Tartu, Estonia; 2 Estonian Biocentre, Genotyping Core Facility, Tartu, Estonia; 3 Estonian Genome Project, University of Tartu, Tartu, Estonia; 4 OÜ BioData, Tartu, Estonia; 5 Department of Clinical Neurology, Medical University of Vienna, Vienna, Austria; 6 Department of Medical Genetics, Medical University of Sofia, Sofia, Bulgaria; 7 Department of Biology and Medical Genetics, Cystic Fibrosis Centre, University Hospital Motol and 2nd School of Medicine, Charles University Prague, Prague, Czech Republic; 8 Department of Neonatology, Clinic of Obstetrics and Gynecology, University Hospital Motol and 2nd School of Medicine, Charles University Prague, Prague, Czech Republic; 9 Wellcome Trust Sanger Institute, Cambridge, UK and the Institute of Molecular Medicine, Biomedicum Helsinki, Helsinki, Finland; 10 Institute for Molecular Medicine Finland (FIMM) and National Institute for Health and Welfare, Helsinki, Finland; 11 Commissariat à l'Energie Atomique, Institut Genomique, Centre National de Génotypage, Evry, France; 12 Fondation Jean Dausset-CEPH, Paris, France; 13 UMR U557 Inserm, U1125 Inra, Cnam, Paris 13, Paris, France; 14 PopGen Biobank, University Hospital Schleswig-Holstein, Campus Kiel, Kiel, Germany; 15 Institute of Human Genetics, Helmholtz Zentrum München, German Research Center for Environmental Health, Neuherberg, Germany; 16 Institute of Human Genetics, Technische Universität München, Klinikum rechts der Isar, Munich, Germany; 17 Institute of Epidemiology, Helmholtz Zentrum München, German Research Center for Environmental Health, Neuherberg, Germany; 18 Institute of Medical Informatics, Biometry and Epidemiology, Ludwig-Maximilians-Universität, Munich, Germany; 19 Department of Medical Genetics and Child Development, University of Pécs, Pécs, Hungary; 20 Division of Genetics and Cell Biology, San Raffaele Research Institute, Milano, Italy; 21 Medical Genetics, Department of Reproductive Sciences and Development, IRCCS-Burlo Garofolo, University of Trieste, Trieste, Italy; 22 Medical Genetics, Institute for Maternal and Child Health - IRCCS “Burlo Garofolo”, Trieste, Italy; 23 Latvian Biomedical Research and Study Center, Riga, Latvia; 24 Department of Human and Medical Genetics, Vilnius University, Vilnius, Lithuania; 25 International Hereditary Cancer Center, Pomeranian Medical University, Szczecin, Poland; 26 Institute of Molecular Genetics, Russian Academy of Science, Moscow, Russia; 27 Center for Genomic Regulation (CRG-UPF) and CIBERESP, Barcelona, Spain; 28 Rheumatology Research group, Vall d'Hebron University Hospital Research Institute, Barcelona, Spain; 29 Department of Genetic Medicine and Development, University of Geneva Medical School, Geneva, Switzerland; Smithsonian Institution National Zoological Park, United States of America

## Abstract

Using principal component (PC) analysis, we studied the genetic constitution of 3,112 individuals from Europe as portrayed by more than 270,000 single nucleotide polymorphisms (SNPs) genotyped with the Illumina Infinium platform. In cohorts where the sample size was >100, one hundred randomly chosen samples were used for analysis to minimize the sample size effect, resulting in a total of 1,564 samples. This analysis revealed that the genetic structure of the European population correlates closely with geography. The first two PCs highlight the genetic diversity corresponding to the northwest to southeast gradient and position the populations according to their approximate geographic origin. The resulting genetic map forms a triangular structure with a) Finland, b) the Baltic region, Poland and Western Russia, and c) Italy as its vertexes, and with d) Central- and Western Europe in its centre. Inter- and intra- population genetic differences were quantified by the inflation factor lambda (λ) (ranging from 1.00 to 4.21), fixation index (F_st_) (ranging from 0.000 to 0.023), and by the number of markers exhibiting significant allele frequency differences in pair-wise population comparisons. The estimated lambda was used to assess the real diminishing impact to association statistics when two distinct populations are merged directly in an analysis. When the PC analysis was confined to the 1,019 Estonian individuals (0.1% of the Estonian population), a fine structure emerged that correlated with the geography of individual counties. With at least two cohorts available from several countries, genetic substructures were investigated in Czech, Finnish, German, Estonian and Italian populations. Together with previously published data, our results allow the creation of a comprehensive European genetic map that will greatly facilitate inter-population genetic studies including genome wide association studies (GWAS).

## Introduction

Over the last few years, the number of genome-wide association studies GWAS has increased markedly and, in concert, these efforts have led to the identification of a large number of new susceptibility loci for common multi-factorial disorders [1]. The underlying technology is developing rapidly and is currently moving from the use of high density SNP arrays towards medical re-sequencing of large genomic regions. Given this development, the availability of thoroughly phenotyped patient and control samples is becoming even more important. Furthermore, due to the small effect sizes that characterize susceptibility genes for multi-factorial traits, potentially successful GWAS rely on large sample number, with additional pressure put on the quality of samples [2]. In reality, however, there will be only very few cohorts comprising 10,000 or even more samples (www.p3gconsortium.org). Exceptions include, for example, the DeCODE studies in Iceland (www.decode.com) and the EPIC (European Prospective Investigation into Cancer and Nutrition) cohort (http://epic.iarc.fr). Collaborations involving diverse sample collections are therefore essential and efforts in this field are promising, for example the establishment of the Biobanking and BioMolecular Resource Infrastructure (www.bbmri.eu). With cohorts from different countries or even from different sites within the same country being used for genetic epidemiological research, the problem of confounding by population stratification has to be addressed. Fortunately, with the vast amount of the genome-wide data available, the actual extent and relevance of population genetic differences can be clarified with high confidence for most commonly used SNP sets.

Confounding by population stratification has been extensively studied in the past [3]. Heterogeneity between studied samples can give false-positive results in association studies, as the association with the trait may by the result of the systematic ancestry difference in allele frequencies between groups [4]. Three main approaches have been proposed so far to capture population genetic differences analytically, namely a) Bayesian clustering [5], b) principal component (PC) analysis [6] and c) multidimensional scaling (MDS) analysis based upon genome-wide identity-by-state (IBS) distances [7]. With the recent availability of high density SNP data, PC and MDS methodologies have become increasingly popular because they require less computing power and have higher discriminatory power than Bayesian analysis for closely related (e.g. European) populations [8]. Therefore, PC analysis is more widely used in the literature. Examples of its recent use are provided by the analysis of high density microarray SNP data at either a global level [9], [10] or, in greater detail, for selected European populations [11]–[15] or within a single country [16]–[18].

In Europe, PC analysis has revealed the strongest genetic differentiation between the northwest and southeast of the continent. The first PC accounts for approximately twice as much of the genetic variation as PC2 [12], [13], [15]. In addition, Price et al. (2008) have shown in their study of US Americans of European descent that the consideration of three clusters of individuals, which roughly corresponded to Northwest Europe, Southeast Europe and Ashkenazi Jewish ancestry, may be sufficient to correct for most of the population stratification affecting genetic association studies. However, the extent to which the results of PC analysis reflect the true underlying genetic map of Europe is critically dependent upon the choice of populations analyzed. Optimal coverage of European populations has not been achieved so far and still represents a goal for future collaborative studies. At present, however, it appears essential that the peripheral populations of Europe or those with a strong founder effect in particular must not be left out of studies aiming at the construction of a continent-wide genetic map.

Here, we present an analysis of more than 270,000 SNPs, genotyped with the Illumina 318K/370CNV chips, on 3,112 individuals across 16 European countries (comprising 19 different samples). Our focus has been on the Baltic region and Eastern Europe since these regions have not been studied in much detail before. The results suggest that geographically adjacent populations overlap partly according to the PC analysis forming four subgroups. Consideration of the inflation factor lambda (λ) [19] further indicates that the loss of power would be minimal when performing and adjusting genetic association studies within these groups.

## Results

In order to investigate in detail the genetic structure of the Baltic countries and neighbouring North-Eastern Europe, whole genome genotyping was undertaken for over 1,000 Estonians and additional individuals from Bulgaria, the Czech Republic, Hungary, Latvia, Lithuania, Poland and Russia, using the Illumina Human370CNV chip. In addition, raw genotyping data were obtained from Scandinavia and other Western and Northern European countries (Table 1). From samples with >100 individuals available (Table 1), a sub-set of 100 individuals was chosen at random for subsequent analyses in order to minimize sample size effects. In all instances, the inflation factor λ as computed for the complete data set *versus* the random sub-set was close to unity, indicating that the latter sets were representative of the entire samples. In total, genotypes of 273,464 SNPs from 1,564 individuals were included in the statistical analyses.

**Table 1:** Studied samples.

Country	Code	# of individuals	# of individuals after QC	# randomly selected 100 individuals	Illumina genotyping assay
Austria (Vienna)	AT	88	87	87	CNV370^a^
Bulgaria	BG	48	47	47	CNV370^b^
Czech Republic (Prague and Moravia)	CZ	94	89	89	CNV370^b^
Estonia	EE	1090	966	100	CNV370^b^
Finland (Helsinki)	FI (HEL)	100	100	100	CNV370^a^
Finland (Kuusamo)	FI (KUU)	84	79	79	CNV370^a^
France (Paris)	FR	100	100	100	HumHap300^a^
Northern Germany (Schleswig-Holstein)	DE (N)	210	206	100	HumHap300^a^
Southern Germany (Augsburg region)	DE (S)	473	468	100	CNV370^a^
Hungary	HU	50	49	49	CNV370^b^
Northern Italy (Borbera Valley)	IT (N)	96	53	53	CNV370^a^
Southern Italy (Region of Apulia)	IT (S)	95	57	57	CNV370^a^
Latvia (Riga)	LV	95	87	87	CNV370^b^
Lithuania	LT	95	90	90	CNV370^b^
Poland ((West-Pomerania)	PL	48	45	45	CNV370^b^
Russia (Andeapol district of Tver region)	RU	96	94	94	CNV370^b^
Spain	ES	200	194	100	HumHap300^a^
Sweden (Stockholm)	SE	100	87	87	HumHap300^a^
Switzerland (Geneva)	CH	216	214	100	HumHap550^a^
Total		3378	3112	1564	

a Raw data provided.

b Genotyped at Estonian Biocentre.

The HapMap data was used for valuation of our results and showing the genetic distance from other continents. The HapMap data included four populations: CEU – U.S. Utah residents with ancestry from Northern and Western Europe, YRI - the Yoruba people of Ibadan, Nigeria, CHB – Han Chinese from Beijing, and JPT – Japanese from Tokyo, in total of 203 individuals.

### Minor allele frequency (MAF)

PLINK was used to compute the minor allele frequencies (MAF) using all the 273,464 SNPs that passed the quality control (QC) procedures. Since the Estonian biobank sample (www.geenivaramu.ee) has been part of several previous GWAS, it was interesting to compare the MAF spectrum seen particularly in Estonia with that of other populations. The correlation coefficient r^2^ obtained varied markedly, from 0.9247 for Latvia and 0.8913 for Finland (Helsinki) to 0.7312 for Southern Italy. In order to examine the extent and likely impact of MAF differences between the studied populations in general, we next examined LD structure, undertook PCA, and calculated fixation indexes F_st_ and inflations factor λ (see below).

### Linkage disequilibrium (LD) structure

**Figure 1:**
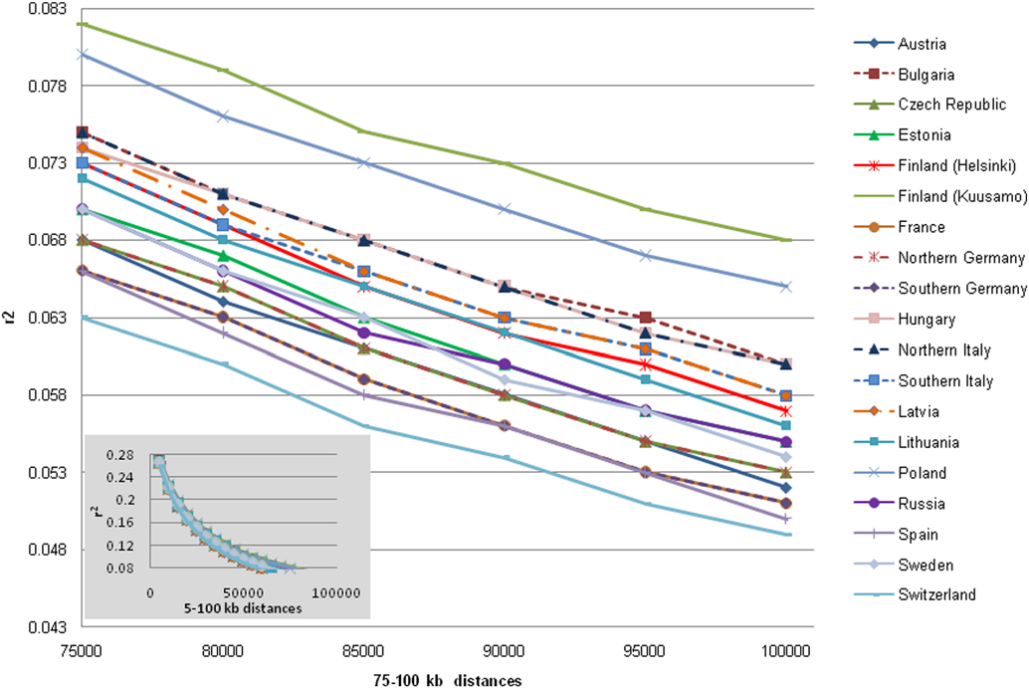
Genome-wide LD pattern (based on 273,464 SNPs), measured by average r^2^, at 5 kb to 100 kb inter-marker distance. Averages were obtained within distance categories according of size 5 kb, i.e. 0–5 kb, 5–10 kb, etc.

Pair-wise LD between SNPs was measured by means of the r^2^ statistics (see Methods). Genome-wide, average r^2^ ranged from 0.24 to 0.28 at smaller distances (5 kb), and decreased to between 0.05 and 0.07 at larger distances (100 kb), depending upon population. Above 75 kb the cohorts started to diverge reflecting the LD extinction towards the north (Figure 1), although the difference was not statistically significant (one-tailed t-test, p-value≤0.05 was considered as statistically significant).

### Principal component (PC) and multidimensional scaling (MDS) analysis

**Figure 2:**
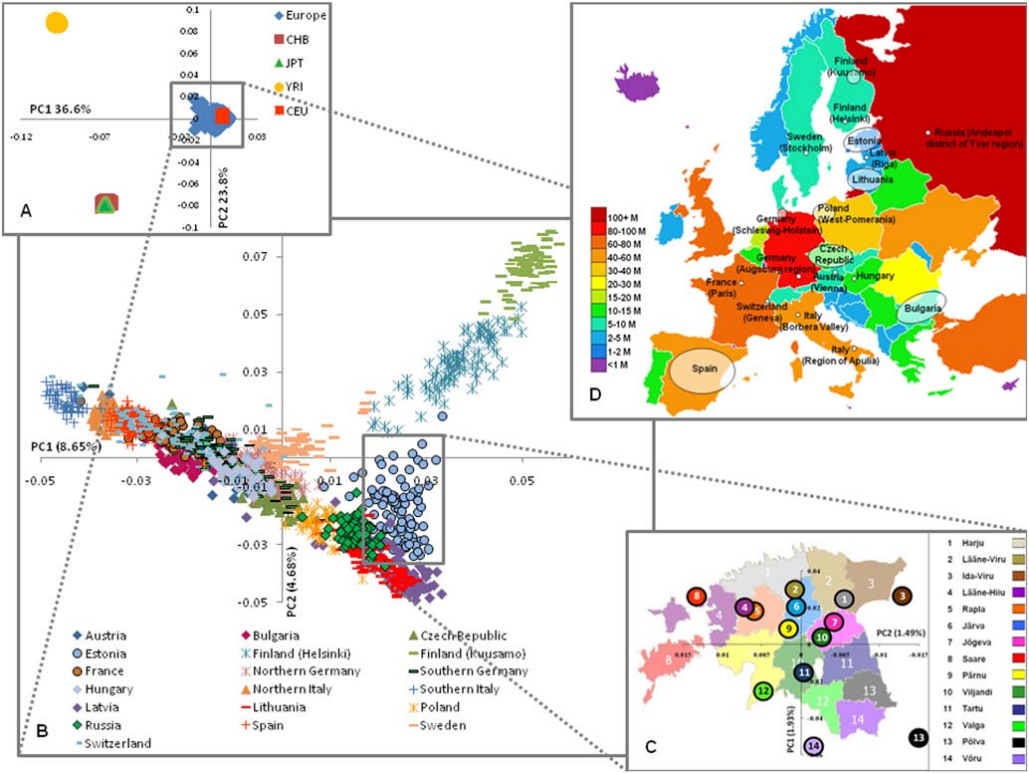
The European genetic structure (based on 273,464 SNPs). Three levels of structure as revealed by PC analysis are shown: A) inter-continental; B) intra-continental; and C) inside a single country (Estonia), where median values of the PC1&2 are shown. D) European map illustrating the origin of sample and population size. CEU - Utah residents with ancestry from Northern and Western Europe, CHB – Han Chinese from Beijing, JPT - Japanese from Tokyo, and YRI - Yoruba from Ibadan, Nigeria.

PC analysis has been used in most previous studies of the European genetic structure. Here, PC analyses were performed using EIGENSOFT with default parameters. In total, 1,564 individuals plus 203 HapMap members and 266,356 autosomal SNPs were used as the input dataset. After the removal of outliers, 1,539 individuals (or 1,742 including the HapMap members) remained (Table S1). The first PC explains 8.7% of the genetic variance, the second PC explains 4.9%; all other PC explained much smaller fractions demonstrating that the Europe is genetically quite uniform. If we add African and Asian HapMap populations to European samples, the two first PCs describe 36.6% and 23.8% of the genetic variance (Figure 2). At a more detailed level, however, several distinct regions can be distinguished within Europe: 1) Finland, 2) the Baltic region (Estonia, Latvia and Lithuania), Eastern Russia and Poland, 3) Central and Western Europe, and 4) Italy, with the southern Italians being more “distant” (Figure 2). PC analysis of the 1,026 Estonians revealed the fine-structure of this population, with the first two PCs describing 1.9% and 1.5% of the genetic variance, respectively. The spread of Estonian individuals is relatively wide as the subregions overlap on individual level, but the median value of PCs, calculated for each county show a remarkable correlation with the regional map of Estonian geography (Figures 2 and S1). PC analysis of genome-wide SNP genotypes is therefore capable of highlighting both global and minute intra-population genetic differences (Figure 2).

As expected, MDS analyses of the data with PLINK yielded a scatter plot of the two first dimensions that looked very similar to that generated by PC analyses (Figure S2).

The twenty-two (11 SNPs for the first PC and 11 SNPs for the second) most variable SNPs presented as default output of the EIGENSOFT analysis are listed on Table S3. These SNPs have significantly different allele frequencies between studied populations and correspond to the largest eigenvalues of the first two PCs explaining the most variance.

### Fixation index (F_st_)

Pair-wise F_st_ values between samples were calculated using EIGENSOFT. F_st_ values indicate how much of the genetic variability between individuals from different populations is due to population affiliation. In our study, F_st_ was found to correlate considerably with geographic distances (r^2^ = 0.382, p-value≪0.01). Values ranged from ≤0.001 for neighbouring populations to 0.023 for Southern Italy and in a young subisolate of Finland (Kuusamo) (Table S2). The F_st_ distances between HapMap CEU sample and the other samples also correlated with geographic distance (r^2^ = 0.291, p-value<0.01). The German population sample showed zero F_st_ with the CEU sample whereas the Finns from Kuusamo and the southern Italians were most different from them (F_st_ = 0.013 and 0.008, respectively) (Table S2). Pair-wise F_st_ values for CEU and either Latvians, Lithuanians, Estonians or western Russians were intermediate (0.006, 0.005, 0.004 and 0.004, respectively).

Two or more samples were available from several countries which allowed us to measure the intra-population variability by F_st_. Mean F_st_ was 0.001 for the 14 Estonian counties, 0.005 for Finland, 0.000 for Germany and 0.005 Italy (each with two samples), and 0.007 for the HapMap CHB and JPT samples. Multi-sample populations were taken from the final PC map to demonstrate the substructure of the populations (Figure S3).

Pair-wise F_st_ of the four HapMap samples (203 individuals in total) were as follows: Europeans (CEU) – Africans (YRI) 0.153; Europeans (CEU) – Japanese (JPT) 0.111; Europeans (CEU) – Chinese (CHB) 0.110; Africans (YRI) – Chinese (CHB) 0.190; Africans (YRI) - Japanese (JPT) 0.192; Chinese (CHB) – Japanese (JPT) 0.007.

Using Barrier 2.2 software, we also correlated geographic and genetic distances as measured by pair-wise F_st_ and great-circle coordinates of capitals or the city where an individual population sample had been recruited, respectively. The results overlapped with previous findings in that the first barrier was seen between Finland and all other samples, a second barrier separated Southern Italy from the remainder, a third was found between Western Russia, Poland and Lithuania on the one hand, and Bulgaria on the other, a fourth was seen between Kuusamo and Helsinki, and a fifth was between the Baltic region and Poland on the one hand, and Sweden on the other (Figure S4).

### Inflation factor lambda (λ)

Table 2 lists the pair-wise inflation factor λ between studied samples. The inflation factor λ was calculated with the method of the Genomic Control [19]. We assumed λ to be constant across the genome and λ was estimated as the median of the observed chi-square statistics divided by the median of the central chi-square distribution with 1 degree of freedom (i.e. 0.456). This factor was found to range from unity (between the samples from the same country) to 4.21 (between Spain and the Kuusamo region). The overall average λ value was 1.82; in separate clusters it amounted to 1.23 (Baltic Region, Western Russia and Poland), 1.54 (Italy and Spain), 1.22 (Central and Western Europe), and 1.86 (Finland), respectively. The correlation coefficient between geographic distance and λ was r^2^ = 0.386 (p-value≪0.01). This value is probably an underestimate of the European-wide relationship due to the inclusion of the Kuusamo and Geneva samples. One is an isolate and the other is a highly heterogeneous international metropolis. The λ values between CEU and the other samples (Table 2) were smaller than those obtained using the Northern German sample as a reference, chosen as the nearest to the origin of CEU sample, and the correlation between geography and λ with CEU was only r^2^ = 0.251 (p-value 0.017). Both results probably reflect the higher genetic variability in the CEU sample.

**Table 2:** Number of significant (p<0.05) SNPs (based on the 273,464 markers) between populations (≤100 samples from every population), using Bonferroni corrected trend test and the inflation factor λ from the genomic control.

# of significant SNPs/Inflation factor λ	Austria	Bulgaria	Czech Republic	Estonia	Finland (Helsinki)	Finland (Kuusamo)	France	Northern Germany	Southern Germany	Hungary	Northern Italy	Southern Italy	Latvia	Lithuania	Poland	Russia	Spain	Sweden	Switzerland	CEU
Austria	-	0	0	2	67	468	0	0	1	0	2	25	8	8	0	2	0	1	0	1
Bulgaria	1.14	-	0	9	68	293	0	8	0	0	0	0	11	13	0	6	2	24	0	14
Czech Republic	1.08	1.21	-	1	47	498	0	0	0	0	2	32	2	2	0	0	3	4	0	1
Estonia	1.58	1.70	1.42	-	8	229	30	4	3	1	84	288	0	0	0	1	155	6	45	20
Finland (Helsinki)	2.24	2.19	2.20	1.71	-	6	190	48	73	20	253	630	85	114	4	41	515	10	230	21
Finland (Kuusamo)	3.30	2.91	3.26	2.80	1.86	-	978	492	593	170	758	1470	598	567	109	410	1620	252	988	215
France	1.16	1.22	1.35	2.08	2.69	3.72	-	1	0	0	2	23	85	37	3	16	0	1	0	0
Northern Germany	1.10	1.32	1.15	1.53	2.17	3.27	1.25	-	0	0	20	79	12	5	0	12	12	0	4	0
Southern Germany	1.04	1.19	1.16	1.70	2.35	3.46	1.12	1.08	-	0	3	34	27	17	4	4	2	2	0	0
Hungary	1.04	1.10	1.06	1.41	1.87	2.68	1.16	1.11	1.08	-	0	4	7	4	0	1	2	18	0	9
Northern Italy	1.49	1.32	1.69	2.42	2.82	3.64	1.38	1.72	1.53	1.42	-	0	118	93	1	42	2	33	0	25
Southern Italy	1.79	1.38	2.04	2.93	3.37	4.18	1.68	2.14	1.85	1.63	1.54	-	337	277	22	133	3	117	3	51
Latvia	1.85	1.86	1.62	1.24	2.31	3.33	2.40	1.84	1.20	1.58	2.64	3.14	-	0	0	0	247	33	122	22
Lithuania	1.70	1.73	1.48	1.28	2.33	3.37	2.20	1.66	1.84	1.46	2.48	2.96	1.20	-	0	0	198	28	67	15
Poland	1.19	1.29	1.09	1.17	1.75	2.49	1.44	1.18	1.23	1.14	1.75	1.99	1.26	1.20	-	0	6	5	1	3
Russia	1.47	1.53	1.27	1.21	2.10	3.16	1.94	1.49	1.58	1.28	2.24	2.68	1.32	1.26	1.18	-	79	27	24	23
Spain	1.41	1.30	1.63	2.54	3.14	4.21	1.13	1.62	1.40	1.32	1.42	1.67	2.82	2.62	1.66	2.32	-	38	0	16
Sweden	1.21	1.47	1.26	1.49	1.89	2.87	1.38	1.12	1.21	1.22	1.86	2.28	1.89	1.74	1.30	1.59	1.73	-	23	0
Switzerland	1.19	1.13	1.37	2.16	2.77	3.83	1.10	1.36	1.17	1.16	1.36	1.54	2.52	2.29	1.46	1.20	1.16	1.50	-	14
CEU	1.12	1.29	1.21	1.59	1.99	2.89	1.13	1.06	1.07	1.13	1.56	1.84	1.87	1.74	1.28	1.56	1.34	1.09	1.21	-

CEU - Utah residents with ancestry from Northern and Western Europe.

The high level of genetic homogeneity in Europe was again highlighted by the λ values calculated between the four HapMap samples (data not shown), which ranged from 21.56 (YRI vs JPT) via 13.27 (CEU vs CHB) to 1.77 between CHB and JPT. The λ value between the African and European samples was slightly smaller than that between the African and Asian samples.

### Marker-wise significance test

Marker-wise significance test was performed in order to assess the allelic distribution in pair-wise comparison of studied cohorts (CEU sample was not included) (Table 2). After applying Bonferroni correction (based on 273,464 markers which equals with the p<1.8×10^−7^) 48 out of 171 of those modeled case-control association analysis between current populations did not reveal any significant hits. Although, in total 16,240 significant hits were identified, while the highest number was 1,620 between Kuusamo and Spain (if Finnish and Italian samples were left out, only comparison with Spanish sample revealed more than 100 markers in single comparison). The average number was 90.4 SNPs and after exclusion of outliers comprising Southern Italy, Kuusamo, Northern Italy and Helsinki data (as the number of significant SNPs was times higher when comparison with Italian and Finnish cohorts) the average decreased to 80.0; 23.0; 21.1 and 10.1 SNPs, respectively. The total number of loci that had a “significant SNP” was 2,263. In order to decrease the amount of loci and identify the meaningful hits, only the loci which had at least two significant hits in at least two pair-wise comparisons were considered, thereby decreasing the total number to 594 loci. Only 18 of those arose from comparisons between other populations than Italy or Finland (Table S4).

## Discussion

Studies of mitochondrial DNA (mtDNA) have suggested substantial genetic homogeneity of European populations [20], with only a few geographic or linguistic isolates appearing to be genetic isolates as well [21]. On the other hand, analyses of the Y chromosome [22], [23] and of autosomal diversity [24] have shown a general gradient of genetic similarity running from the southeast to the northwest of the continent.

In the present study using autosomal SNPs and high density genotyping, we have focused on the genetic structure of the Baltic, Finnish and other North-Eastern European populations, while populations from Western and Southern Europe were included mainly for comparison (Figure 2). Overall the samples under investigation have a large geographic coverage, ranging from Spain and Italy, through the Baltic to Finland and Western Russia. Previous studies have focused upon the genetic structure in Central and Western Europe [11]–[13], Northern Europe [17], [25] or studied US Americans of European and Ashkenazi-Jewish descent [14], [15].

Genome-wide analyses presented here have revealed, as expected, more extensive LD in isolated populations than in outbred populations. It can be presumed that the average r^2^ value, particularly at larger inter-marker distances, reflects the extent of panmixia in a population. Indeed, the Kuusamo sample, a population isolate that was established from a small number of founders only 300 years ago, had the highest r^2^ irrespective of distance in our and previous studies [26]. At the other end of the scale was Geneva, one of the most cosmopolitan cities in Europe, which yielded the lowest r^2^ values. Thus, our data corroborate earlier suggestions that the amount of LD that persists over time is markedly reduced in more admixed populations [27]. Surprisingly, the Polish cohort showed a similar LD pattern as the Kuusamo population, which is probably reflecting the homogeneity of the Polish population. Here the similarity could be attributed to the founder effect or admixture as the Polish sample comes from West Pomerania, a region that was repopulated after the Second World War, after the expulsion of the German population, with other people from (Eastern Poland) and also some Ukrainians. Small sample size (n = 45) does not provide a sufficient explanation for this finding because the Hungarian and Bulgarian samples were also similar in size (Table 1), but gave LD patterns distinct from the Polish and Kuusamo samples (Figure 1).

PC analysis yielded a genetic map where the first two PCs highlight the genetic diversity corresponding to the Northwest to Southeast gradient and position the populations according to their approximate geographic origin. Our genetic map shows slightly different tendency from previously published ones in that the scatter plot takes the form of a triangle, with the Finnish, Baltic and Italian samples as its vertexes, and with Central Europe residing in its centre. The two PCs explain 8% and 4% of the genetic variability in the samples, which is almost twice as much as in previous European-based studies. This increase is likely due to the fact that the geographic coverage in our study has been broader and that our data captured more genetic variability (Figure 2).

Interestingly, PC analysis was also capable of highlighting intra-population differences, such as between the two Finnish and the two Italian samples, respectively. A low level of intra-population differentiation in Germany has been reported previously [18], and was confirmed here. In addition, we detected intra-population differences within the Czech and Estonian samples (Figure S3). In the case of the Czech, two samples were available: Prague and Moravia. Although their pair-wise F_st_ was virtually zero, the median values of PCs for the two samples sets are different. This is explicable by the fact that Moravia has a long shared history with the remainder of the Czech Republic, but is nevertheless separated from the rest of the country by the Czech-Moravian highlands, which in the past hindered stronger intermixing.

Estonia is a small country with no geographic barriers and its Estonian population is merely one million. In order to study the genetic structure of Estonia in more detail, all Estonian individuals were grouped here by their county of birth. Then, PCA was performed and the mean values of the two first PC of the counties were plotted onto the Estonian regional map (Figure 2). Surprisingly, the resulting genetic map correlates almost perfectly with the geographic map, although Estonia is only 43,400 km^2^ in size, and the mean area of a county only 2,900 km^2^. Thus, fine-scale genetic difference can be revealed by PC analysis, and the results can be useful for identification of the distant relatives.

Barrier analysis revealed genetic barriers between Finland, Italy and other countries, as has been described before [12]. Interestingly, barriers could be demonstrated within Finland (between Helsinki and Kuusamo) and Italy (between northern and southern part). Another barrier emerged between the Eastern Baltic region and Sweden, but not between the Eastern Baltic region and Poland (Figure S4). The barrier between Bulgaria and Western Russia, Poland and Lithuania may have arisen due to the fact that several populations are missing in between those countries. It has been shown previously that the populations of central European background are less differentiated genetically, whereas the Finns exhibit a more homogeneous population structure with decreased genetic diversity [17], [25].

**Figure 3:**
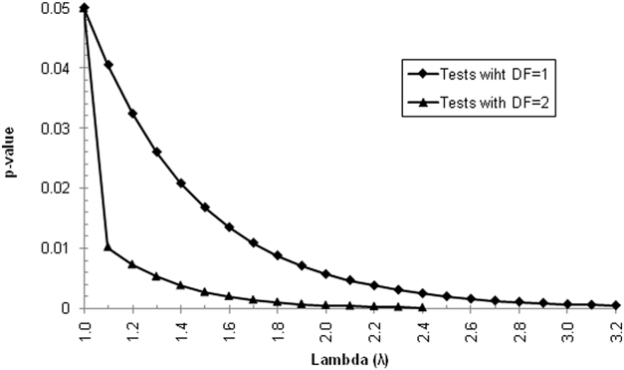
Impact of inflation factor λ upon the required significance of disease-gene association. The graph shows the highest p-value that would stay below 0.05 after correction using a given λ in the Genomic Control approach for two scenarios: 1) the decrease of chi-square statistics in a test with 1 degree of freedom (e.g. Allelic, Additive, Dominant, Receive), and 2) in a test with two degrees of freedom (e.g. Genotypic).

In GWAS using large numbers of markers, multiple testing correction becomes an important issue, and a genome-wide significance threshold of p<5×10^−7^ has been proposed [16]. At the same time, adjustment for population stratification can decrease the necessary level of nominal significance even further. This can be illustrated, for example, by adopting the Genomic Control approach [19] where the factor λ by which the chi-squared statistic is inflated by confounding is first estimated from the null loci and correction is then applied by dividing the actual association chi-square statistic by λ. Figure 3 illustrates the effect that this procedure would have by showing, for each possible λ, the highest p-value that stays below 0.05 after correction. Two scenarios are presented: 1) tests with 1 degree of freedom (Allelic, Additive, Dominant and Receive) and 2) tests with 2 degrees of freedom (Genotypic). When λ = 1.5 (which would be common if patients and controls came from different European countries) (Table 2), the original p-value must be approximately three times lower than 0.05. For geographically distant samples, the necessary reduction may be by a factor of up to 500, as would be the case with Kuusamo and Southern Italy. Interestingly, λ values with respect to other samples are smaller for CEU (originating mostly from Northern Germany, Netherlands and Belgia [12]) than for Northern Germany. This is probably due to the higher genetic variability in the CEU sample, ancestry of which is from a mixture of several different populations and therefore the CEU sample is a better reference for European population than a single population. It should be pointed out that any adjustment for stratification does inflate the multiple testing correction so that, if genetically distant case and control samples are compared in an association study, the genome-wide significance threshold in some cases would even be as low as p<1×10^−10^.

From our results, conclusions can be drawn as to which European populations can be combined in GWAS, considering the pair-wise calculations of inflation factor λ and F_st_ values, although meta-analyses may often be a more appropriate option [28], [29].

Marker-wise significance test for allelic differences in pair-wise comparisons between the studied samples resulted in 2,263 loci. As our sample included some genetically and geographically distant cohorts (Finns and Italians) where the strong founder effect and isolation driven genetic drift has changed respective allele frequencies, therefore only loci that were present in non-Italian and non-Finnish comparisons were considered. This step decreased the number of significantly different loci to 18 (Table S4). Four genes were within LCT loci (haplotype block covering more than 1 Mb [30]) and it has been shown, that LCT region differentiates European populations [11], but also within a given population [16]. Three genetically most variable SNPs revealed by PC analysis represented the same loci also present in the previously mentioned list of 18 loci.

In conclusion, we have described the European genetic structure by three different measures: the inflation factor λ, F_st_ and PC. As a result, according to the first two PCs, individuals from the same geographic origin cluster together and form a genetic map where four areas could be identified: 1) Central and Western Europe, 2) the Baltic countries, Poland and Western Russia, 3) Finland, and 4) Italy. If not corrected for the inter-population differences would affect the significance of disease-gene associations. A detailed description of the European population structure has consequences and implications for the design of future GWAS, particularly regarding sample size and choice of controls. As a matter of fact, the knowledge of genetic distances between different populations is helpful in defining which biobanks could sensibly contribute samples and data to GWAS.

## Materials and Methods

### Ethics Statement

The study was approved by the Ethics Review Committee on Human Research of the University of Tartu (166/T-21, 17.12.2007). Written informed consent for participation was obtained from all study subjects.

### Samples

Samples are described in detail in the supplementary methods section (Text S1). The studied 3,112 individuals representing a total of 19 cohorts (Czech Republic samples were used as one in all analyses except from the inter-population structure analyses) samples from 16 countries: Austria (Vienna), Bulgaria (entire country), Czech Republic (Prague, Moravia and Silesia), Estonia (entire country), Finland (Helsinki, and a young internal subisolate of Kuusamo), France (Paris), Germany (Schleswig-Holstein, Augsburg region), Hungary (entire country), Italy (Borbera Valley, Region of Apulia), Latvia (Riga), Lithuania (entire country), Poland (West-Pomerania), Russia (Andreapol district of the Tver region), Spain (entire country), Sweden (Stockholm) and Switzerland (Geneva) (Table 1).

The HapMap data used in our study comprised four populations, namely CEU - U.S. Utah residents with ancestry from Northern and Western Europe, YRI - the Yoruba people of Ibadan, Nigeria, CHB - unrelated individuals from Beijing, China, and JPT - unrelated individuals from Tokyo, Japan. HumanHap300 (v1-0.0) genotypes were downloaded from Illumina iControlDB 1.1.2 (www.illumina.com/pages.ilmn?ID=231), comprising a total of 203 individuals. For the CEU and YRI samples, only parents were used.

### Genotyping

For the samples from Bulgaria, Czech Republic, Estonia, Hungary, Latvia, Lithuania, Poland and Russia, genotyping was performed at the Estonian Biocentre (Tartu, Estonia) according to the manufacturer's instructions, using the Illumina Human370CNV-duo chips.

Additional raw genotyping data were obtained for the samples from Austria, Finland, Southern Germany (Augsburg region) and Italy for Illumina Human370CNV-duo, from France, Northern Germany (Schleswig-Holstein), Spain and Sweden for HumanHap300-duo, and from Switzerland for HumanHap550 data.

Systematic quality control (QC) was applied to all genotypes generated at the Estonian Biocentre. Duplicates from the Estonian sample were used to assess genotyping reproducibility, i.e. every 40^th^ individual was duplicated and the mean discordance per SNP between pairs of individuals was found to be less than 1 in 5000 (0.0002%). The per individual call rate had to be at least 95% for individuals to be included into subsequent analyses. The number of individuals before and after QC is shown in detail in Table 1.

Only the genotypes for those 311,226 SNPs that were typed in all 3,378 individuals were included in subsequent computational analyses. Closely related individuals were identified using estimation of the proportion of the genome shared identical by descent (IBD), and the relative with the lower call rate was removed. Inbreeding coefficient F was assessed in order to detect potential DNA contamination. SNPs found to be out of Hardy-Weinberg equilibrium at p<10^−5^, or missing more than 1% of genotypes, or with a minor allele frequency <0.01 were removed from the dataset [16]. The total rate of genotyping calls in the remaining individuals was 0.995. After QC, 273,454 SNPs remained (from 3,112 individuals), including 203 HapMap individuals that increased the overall sample size to 3,315. All QC procedures were conducted with Illumina's BeadStudio (www.illumina.com) and the PLINK software [7].

### Statistical analysis

Pair-wise LD was measured by r^2^ for all SNPs less than 100 kb apart using the Haploview software [31]. A custom Perl script was used to categorize r^2^ according to inter-marker distance (0–5 kb, 5–10 kb etc.) and mean r^2^ was calculated for each category. The significance of the mean r^2^ values between cohorts was tested with the one-tailed t-test and p-value≤0.05 was considered as statistically significant.

Principal component (PC) analysis was performed and F_st_ determined between samples using EIGENSOFT [6] on three sets of samples: 1) HapMap+Europe, 2) Europe, and 3) Estonia alone with individual counties. All analyses were performed with the default parameters. Multidimensional scaling (MDS) analyses were performed with the PLINK software. The marker set was filtered according to pair-wise LD (r^2^ cut-off = 0.2) in order to remove correlated markers. The number of remaining markers was 68,201. All PC values and MDS dimensions were multiplied by −1 to render scatter plots more similar to the geographic distribution of individual origin.

Geographic barriers were computed with the Barrier v2.2 software [32]. For the geographic positioning of samples the great-circle coordinates of the respective capital of the country of origin, or the city where an individual population sample had been recruited was used. The F_st_ pair-wise comparison matrix for genetic and geographic distance was used in barrier analyzes. The geographic location of the CEU sample was approximated by Northern Germany (as shown in the Lao et al. 2008 paper). The geographic distances between the above mentioned cities were used to calculate the correlation coefficient between geography and statistics, like F_st_ and inflation factor λ. Statistical tests were performed in R v2.8.1 (www.R-project.org).

Trend tests were performed in order to identify markers with significant pair-wise allele frequency differences between populations. The resulting p-values were subjected to Bonferroni correction and the significance threshold was set at p<0.05, although the multiple testing which arises from the pair-wise comparisons was not taken into account. The “inflation factor” λ of the Genomic Control method [19] was calculated using HelixTree (Golden Helix, Inc. Bozeman, MT, USA, HelixTree® Software; www.goldenhelix.com).

## Supporting Information

Text S1 Study subjects
(0.04 MB DOC)

Table S1 Sample sizes for principal component analysis of 266,356 SNPs.
(0.05 MB DOC)

Table S2 Pair-wise Fst between European samples.
(0.10 MB DOC)

Table S3 Most variable SNPs in the PC analysis.
(0.07 MB DOC)

Table S4 Top eighteen genetically most variable loci from the pair-wise cohort association analysis. The locus was described by at least two SNPs and was present in at least two pair-wise cohort analyses.
(0.06 MB DOC)

Figure S1 PC map of Estonian counties. Shown are the Estonian samples grouped by county. The great circles mark the median PC values for each county. Counties are colour-coded as shown in the inset.
(0.25 MB TIF)

Figure S2 Multidimensional scaling plot of the studied European individuals.
(0.14 MB TIF)

Figure S3 Population structure within studied populations. The scatter plots of the first two PCs show the level of stratification within A) Czech Republic - north-western part (Czech lands) and south-eastern part (Moravia), plus Austrian samples, B) Germany - northern and southern part, C) Italy - northern and southern part, and D) Estonia - northern and southern part.
(0.15 MB TIF)

Figure S4 Barrier analysis of European gene pool. The analysis was based upon great-circle coordinates of the cities where individual population samples were recruited and pair-wise Fst. The name of the city is indicated in the brackets in the left panel. Lower case letters point the order of the found barriers.
(0.15 MB TIF)
