# Correction: Genetic Structure of Europeans: A View from the North–East

**DOI:** 10.1371/annotation/2849e182-aef5-4e2b-a5ac-0b74b30e5f48

**Published:** 2010-03-26

**Authors:** Mari Nelis, Tõnu Esko, Reedik Mägi, Fritz Zimprich, Alexander Zimprich, Draga Toncheva, Sena Karachanak, Tereza Piskáčková, Ivan Balaščák, Leena Peltonen, Eveliina Jakkula, Karola Rehnström, Mark Lathrop, Simon Heath, Pilar Galan, Stefan Schreiber, Thomas Meitinger, Arne Pfeufer, H-Erich Wichmann, Béla Melegh, Noémi Polgár, Daniela Toniolo, Paolo Gasparini, Pio D'Adamo, Janis Klovins, Liene Nikitina-Zake, Vaidutis Kučinskas, Jūratė Kasnauskienė, Jan Lubinski, Tadeusz Debniak, Svetlana Limborska, Andrey Khrunin, Xavier Estivill, Raquel Rabionet, Sara Marsal, Antonio Julià, Stylianos E. Antonarakis, Samuel Deutsch, Christelle Borel, Homa Attar, Maryline Gagnebin, Milan Macek, Michael Krawczak, Maido Remm, Andres Metspalu

The third author, Reedik Mägi, should be noted as affiliated with: Estonian Biocentre, Tartu, Estonia, and the Wellcome Trust Centre for Human Genetics, University of Oxford, Oxford, United Kingdom.

