# Supplementary material for: Genetic Structure of Europeans: A View from the North–East
Source: PLoS One. 2009 May 8;4(5):e5472. doi: 10.1371/journal.pone.0005472 (PMC2675054; doi:10.1371/journal.pone.0005472)
Supplement: Text S1 — Study subjects (0.04 MB DOC) [file pone.0005472.s001.doc]

**Text S1.** Study subjects

Written informed consent for participation was obtained from all study subjects.

**Austria:**

The donors were recruited via a local blood donation center in Vienna. The samples were collected as controls for various genetic epilepsy studies. To be included, donors had to be of self identified ethnic Austrian origin, and did not have any chronic diseases.

**Bulgaria:**

The Bulgarian samples were selected randomly from healthy individuals through the whole country (with sex ratio 1:1).

**Czech Republic:**

Prague samples were derived from cord blood from physiological newborns from Prague and surrounding Central Bohemian Region (born 10/2007-2/2008), while Moravian / Silesian samples include unaffected relatives of patients treated for infertility and for inherited trombophilia. The latter cohort covers the entire geographic region of the eastern part of the Czech Republic. Male and female ratio was 1:1, as also the Prague and Moravian samples ratio.

**Estonia:**

The 1,090 Estonian samples were selected from 10,317 samples of the Estonian biobank (year 2005). Eighty samples (40 males and 40 females) were selected randomly, according to the place of birth, from each of 13 Estonian counties (Harju, Ida-Viru, Jõgeva, Järva, Lääne-Viru, Põlva, Pärnu, Rapla, Saaremaa, Tartu, Valga, Viljandi, Võru), and 50 samples (25 males and 25 females) were selected from the combined Hiiumaa and Läänemaa counties. Prior to collection approval from the Ethics Committee of the Estonian biobank was obtained.

**Finland:**

The Helsinki samples were collected as part of a larger local project to represent the general Finnish population. Both males and females are included and the selection was not based on any disease/diagnosis/trait. The Kuusamo samples represent the general population in the Kuusamo region (a Finnish isolate on the north-eastern border of the country). The selected individuals have both parents born in Kuusamo. Both males and females are included and the selection was not based on any disease/diagnosis/trait.

**France:**

The French samples are a non-selected cohort from the Paris area. They are part of a larger cohort study (~20000 samples). For the current analysis the genotyping data for the first 50 males and 50 females have been used.

**Northern Germany:**

The Northern German sample represents the county of Schleswig-Holstein (population size: 1.15 million) and has been collected as part of the POPGEN project [33].

**Southern Germany:**

The KORA samples were collected as part of a population-based, epidemiological project, KORA S2000 (Cooperative Health Research in the Region of Augsburg), and represent an urban region in the southern part of Germany with 610,000 inhabitants [34]. The data are from the Survey S4 of the population-based KORA study (Collaborative Health Research in the Region of Augsburg) which was conducted 1999 – 2001 and represent individuals in the age range of 25 to 74 years from Southern Germany [35].

**Hungary:**

The Hungarian samples were collected from unrelated, healthy individuals who represented the average population and did not belong to any minor ethnic group present in the country.

**Northern Italy:**

The Northern Italian samples have been randomly collected from the Borbera Valley. The Borbera Valley is located in Northern Italy, namely the northern part of the Apennines mountains, between Liguria and Piedmont, about 80 km south of Milan. For the current analysis, 96 individuals were selected from the database where all the samples have complete phenotypic data.

**Southern Italy:**

The Italian samples were randomly chosen from those enrolled in a population study named Carlantino Project, which is focused on inhabitants arising from an isolated village at the border between Central and Southern Italy (Province of Foggia, Region of Apulia with 1200 inhabitants).

**Latvia:**

The Latvian samples were selected from a population-based collection that is part of the Genome Database of Latvian Population, the national biobank. Participants were randomly selected from a group of individuals whose nationality and both of their parents’ nationalities were reported as Latvian. Participants were recruited through general practitioners and were required to be older than 18 years, without any chronic disorder; anthropometric measurements (including weight and stature), ethnic, social, environmental information and familial health status were acquired from self-reported questionnaire. The study protocol was approved by Central Medical Ethics Committee of Latvia.

**Lithuania:**

Peripheral blood samples were collected from unrelated individuals from six ethno-linguistic groups of Lithuania. Informed consent and information about birthplace, parents and grandparents were obtained from all donors. Approval was obtained from the Bioethics Committee of Lithuania prior to collection.

**Poland:**

The Polish sample was randomly selected from a population-based collection of adults (with sex ratio 1:1) representing Polish ethnic group from the West-Pomeranian region of Poland. The participants were randomly selected from the patient roles of participating family physicians.

**Russia:**

The Russian samples were obtained from healthy donors from the Andreapol district of the Tver region; all individuals were unrelated to each other and represented the native ethnic group of the region (i.e. they belonged to at least the third generation living in a particular geographic region).

**Spain:**

The Spanish sample comprises a group of 200 individuals described as hypernormal control subjects in the randomized control collection of IRCIS BioBank (Hospital San Joan de Reus, Tarragona, Spain). Individuals were 40 years old, Europeans, and had a 3-generation Spanish origin.

**Sweden:**

Swedish samples were obtained from the GenomEU-twin project, collected from the Stockholm area, and were all female.

**Switzerland:**

The Swiss samples were obtained from the GenCord project (University of Geneva Medical School). The GenCord project holds a collection of cell lines derived from umbilical cords of 200 individuals of Western European origin. Since the collection was performed on full term pregnancies, the samples are homogenous in terms of age and collection procedure.
