# Supplementary material for: Genetic Structure of Europeans: A View from the North–East
Source: PLoS One. 2009 May 8;4(5):e5472. doi: 10.1371/journal.pone.0005472 (PMC2675054; doi:10.1371/journal.pone.0005472)
Supplement: Table S1 — Sample sizes for principal component analysis of 266,356 SNPs. (0.05 MB DOC) [file pone.0005472.s002.doc]

**Table S1.** Sample sizes for principal component analysis of 266,356 SNPs.

| Cohort | # of samples | # of samples after outlier removal |
| --- | --- | --- |
| Austria (Vienna) | 87 | 86 |
| Bulgaria | 47 | 47 |
| Czech Republic (Prague and Moravia) | 89 | 88 |
| Estonia | 100 | 100 |
| Finland (Helsinki) | 100 | 100 |
| Finland (Kuusamo) | 79 | 79 |
| France (Paris) | 100 | 99 |
| Northern Germany (Schleswig-Holstein) | 100 | 100 |
| Southern Germany (Augsburg region) | 100 | 99 |
| Hungary | 49 | 49 |
| Northern Italy (Borbera Valley) | 53 | 48 |
| Southern Italy (Region of Apulia) | 57 | 57 |
| Latvia (Riga) | 87 | 87 |
| Lithuania | 90 | 89 |
| Poland ((West-Pomerania) | 45 | 45 |
| Russia (Andeapol district of Tver region) | 94 | 94 |
| Spain | 100 | 100 |
| Sweden (Stockholm) | 87 | 74 |
| Switzerland (Geneva) | 100 | 98 |
| Subtotal | 1564 | 1539 |
| HapMap samples |  |  |
| CEU | 60 | 60 |
| CHB | 44 | 44 |
| JPT | 44 | 44 |
| YRI | 55 | 55 |
| Total | 1767 | 1742 |

CEU - Utah residents with ancestry from Northern and Western Europe, CHB – Han Chinese from Beijing, JPT - Japanese from Tokyo, and YRI - Yoruba from Ibadan, Nigeria
