# Supplementary material for: Genetic Structure of Europeans: A View from the North–East
Source: PLoS One. 2009 May 8;4(5):e5472. doi: 10.1371/journal.pone.0005472 (PMC2675054; doi:10.1371/journal.pone.0005472)
Supplement: Table S2 — Pair-wise Fst between European samples. (0.10 MB DOC) [file pone.0005472.s003.doc]

**Table S2.** Pair-wise F_st_ between European samples.

|  | Austria | Bulgaria | Czech Republic | Estonia | Finland (Helsinki) | Finland (Kuusamo) | France | Northern Germany | Southern Germany | Hungary | Northern Italy | Southern Italy | Latvia | Lithuania | Poland | Russia | Spain | Sweden | Switzerland |
| --- | --- | --- | --- | --- | --- | --- | --- | --- | --- | --- | --- | --- | --- | --- | --- | --- | --- | --- | --- |
| Bulgaria | 0.001 |  |  |  |  |  |  |  |  |  |  |  |  |  |  |  |  |  |  |
| Czech Republic | 0.000 | 0.002 |  |  |  |  |  |  |  |  |  |  |  |  |  |  |  |  |  |
| Estonia | 0.003 | 0.005 | 0.002 |  |  |  |  |  |  |  |  |  |  |  |  |  |  |  |  |
| Finland (Helsinki) | 0.006 | 0.009 | 0.006 | 0.004 |  |  |  |  |  |  |  |  |  |  |  |  |  |  |  |
| Finland (Kuusamo) | 0.013 | 0.015 | 0.012 | 0.009 | 0.005 |  |  |  |  |  |  |  |  |  |  |  |  |  |  |
| France | 0.001 | 0.002 | 0.002 | 0.005 | 0.008 | 0.015 |  |  |  |  |  |  |  |  |  |  |  |  |  |
| Northern Germany | 0.000 | 0.002 | 0.001 | 0.003 | 0.006 | 0.012 | 0.001 |  |  |  |  |  |  |  |  |  |  |  |  |
| Southern Germany | 0.000 | 0.001 | 0.001 | 0.003 | 0.006 | 0.013 | 0.001 | 0.000 |  |  |  |  |  |  |  |  |  |  |  |
| Hungary | 0.000 | 0.001 | 0.000 | 0.003 | 0.006 | 0.013 | 0.001 | 0.001 | 0.000 |  |  |  |  |  |  |  |  |  |  |
| Northern Italy | 0.004 | 0.003 | 0.005 | 0.010 | 0.013 | 0.020 | 0.003 | 0.005 | 0.004 | 0.004 |  |  |  |  |  |  |  |  |  |
| Southern Italy | 0.006 | 0.004 | 0.007 | 0.013 | 0.016 | 0.023 | 0.005 | 0.008 | 0.006 | 0.006 | 0.005 |  |  |  |  |  |  |  |  |
| Latvia | 0.005 | 0.007 | 0.003 | 0.001 | 0.007 | 0.013 | 0.008 | 0.004 | 0.005 | 0.004 | 0.012 | 0.015 |  |  |  |  |  |  |  |
| Lithuania | 0.004 | 0.006 | 0.002 | 0.001 | 0.007 | 0.013 | 0.006 | 0.003 | 0.004 | 0.003 | 0.011 | 0.014 | 0.001 |  |  |  |  |  |  |
| Poland | 0.001 | 0.003 | 0.001 | 0.001 | 0.006 | 0.012 | 0.003 | 0.001 | 0.002 | 0.001 | 0.007 | 0.010 | 0.002 | 0.001 |  |  |  |  |  |
| Russia | 0.003 | 0.004 | 0.001 | 0.001 | 0.006 | 0.012 | 0.005 | 0.002 | 0.003 | 0.002 | 0.009 | 0.012 | 0.002 | 0.001 | 0.001 |  |  |  |  |
| Spain | 0.002 | 0.002 | 0.003 | 0.008 | 0.011 | 0.017 | 0.001 | 0.003 | 0.002 | 0.002 | 0.003 | 0.005 | 0.010 | 0.009 | 0.005 | 0.007 |  |  |  |
| Sweden | 0.001 | 0.004 | 0.002 | 0.003 | 0.005 | 0.011 | 0.002 | 0.001 | 0.001 | 0.002 | 0.007 | 0.009 | 0.005 | 0.004 | 0.002 | 0.003 | 0.004 |  |  |
| Switzerland | 0.001 | 0.001 | 0.002 | 0.006 | 0.009 | 0.015 | 0.000 | 0.002 | 0.001 | 0.001 | 0.003 | 0.004 | 0.008 | 0.007 | 0.004 | 0.005 | 0.001 | 0.003 |  |
| CEU | 0.001 | 0.003 | 0.001 | 0.004 | 0.006 | 0.013 | 0.001 | 0.000 | 0.000 | 0.001 | 0.005 | 0.008 | 0.006 | 0.005 | 0.003 | 0.004 | 0.002 | 0.001 | 0.002 |

CEU - Utah residents with ancestry from Northern and Western Europe
