# Supplementary material for: Genetic Structure of Europeans: A View from the North–East
Source: PLoS One. 2009 May 8;4(5):e5472. doi: 10.1371/journal.pone.0005472 (PMC2675054; doi:10.1371/journal.pone.0005472)
Supplement: Table S3 — Most variable SNPs in the PC analysis. (0.07 MB DOC) [file pone.0005472.s004.doc]

**Table S3.** Most variable SNPs in the PC analysis.

| Rs # of SNP | Chromosome | Position | Map location | Gene symbol | Additional information |
| --- | --- | --- | --- | --- | --- |
| rs2305142 | 2 | 97742154 | 2q12 | ZAP70 | Neuroactive ligand-receptor interaction |
| rs9881418 | 3 | 144954522 | 3q24 | SLC9A9 |  |
| rs6788064 | 3 | 144959101 | 3q24 | SLC9A9 |  |
| rs6599400 | 4 | 1754823 | 4p16.3 | FGFR3 | Regulation of actin cytoskeleton; MAPK signaling pathway |
| rs2303671 | 5 | 132587849 | 5q31.1 | FSTL4 |  |
| rs2745412 | 6 | 29632380 | 6p21.3 | UBD |  |
| rs10217044 | 8 | 8932162 | 8p23.1 | THEX1 |  |
| rs4388427 | 8 | 99387257 | 8q22.2 | NPAL2 |  |
| rs4978561 | 9 | 115804104 | 9q32 | ZNF618 | Regulation of actin cytoskeleton; MAPK signaling pathway |
| rs4979320 | 9 | 115806098 | 9q32 | ZNF618 | Natural killer cell mediated cytotoxicity; T cell receptor signaling pathway |
| rs3827676 | 9 | 115980840 | 9q32 | COL27A1 |  |
| rs1334059 | 10 | 25571977 | 10p12.1 | GPR158 |  |
| rs543925 | 11 | 115760668 | 11q23.3 | MGC13125 |  |
| rs789327 | 12 | 68716731 | 12q15 | C12orf28 |  |
| rs7960313 | 12 | 84847387 | 12q21 | NTS |  |
| rs1571786 | 13 | 29009933 | 13q12-q14 | SLC7A1 | Determines human skin pigmentation; Ornithine uptake in retinal pigment epithelium |
| rs1120556 | 13 | 87960360 | 13q31.2 | SLITRK5 |  |
| rs9521107 | 13 | 87977213 | 13q31.2 | SLITRK5 |  |
| rs7495174 | 15 | 26017833 | 15q11.2-q12 | OCA2 | Albinism, brown oculocutaneous; Albinism, ocular, autosomal recessive; Albinism, oculocutaneous, type II |
| rs711906 | 15 | 38112983 | 15q15.1 | EIF2AK4 |  |
| rs4775159 | 15 | 57457699 | 15q21-q22 | MYO1E |  |
| rs5007291 | 20 | 56465163 | 20q13.32 | APCDD1L (FLJ90166) |  |
