# Supplementary material for: Genetic Structure of Europeans: A View from the North–East
Source: PLoS One. 2009 May 8;4(5):e5472. doi: 10.1371/journal.pone.0005472 (PMC2675054; doi:10.1371/journal.pone.0005472)
Supplement: Table S4 — Top eighteen genetically most variable loci from the pair-wise cohort association analysis. The locus was described by at least two SNPs and was present in at least two pair-wise cohort analyses. (0.06 MB DOC) [file pone.0005472.s005.doc]

**Table S4.** Top eighteen genetically most variable loci from the pair-wise cohort association analysis. The locus was described by at least two SNPs and was present in at least two pair-wise cohort analyses.

| Gene symbol | Map location | Additional information (KEGG^a^ pathway) | Phenotype |
| --- | --- | --- | --- |
| IFI44 | 1p31.1 |  |  |
| ARHGAP15 | 2q22.2 |  |  |
| CXCR4 | 2q21^b^ | CXCR4 signaling pathway; Pertussis toxin-insensitive CCR5 signaling in macrophage; Selective expression of chemokine receptors during T-cell polarization; HIV-1 defeats host-mediated resistance by CEM15 |  |
| DARS | 2q21.3^b^ |  |  |
| R3HDM1 | 2q21.3^b^ |  | Warburg micro syndrome 1 |
| RAB3GAP1 | 2q21.3^b^ | CXCR4 signaling pathway; Links between Pyk2 and Map kinases; Integrin signaling pathway; Cell to cell adhesion signaling; PTEN dependent cell cycle arrest and apoptosis |  |
| MUC4 | 3q29 |  |  |
| FAM114A1 (LOC92689) | 4p14 |  |  |
| TLR1 | 4p14 |  |  |
| RFXDC1 | 6q22.2 |  |  |
| COX11P | 6p23-p22 |  | Myelokathexis, isolated; Wart, hypogammaglobulinemia, infection, and myelokathexis syndrome |
| GLI3 | 7p13 | Sonic hedgehog (Shh) pathway |  |
| ZNF618 | 9q32 |  |  |
| FLRT2 | 14q24-q32 |  | Acrocallosal syndrome; Greig cephalopolysyndactyly syndrome; Pallister-Hall syndrome; Polydactyly, postaxial, types A1 and B; Polydactyly, preaxial, type IV |
| OCA2 | 15q11.2-q12 |  | Lymphoplasmacytoid lymphoma |
| MCTP2 | 15q26.2 |  | Tietz syndrome; Waardenburg syndrome, type IIA; Waardenburg syndrome/ocular albinism, digenic |
| FLJ44674 | 16q12.1 |  |  |
| APCDD1L (FLJ90166) | 20q13.32 |  |  |
|  |  |  |  |
| ^a^Kyoto Encyclopedia of Genes and Genomes | | |  |
| ^b^LCT region (2q21) | |  |  |
