# Supplementary figures and images for: Genetic Structure of Europeans: A View from the North–East
Source: PLoS One. 2009 May 8;4(5):e5472. doi: 10.1371/journal.pone.0005472 (PMC2675054; doi:10.1371/journal.pone.0005472)

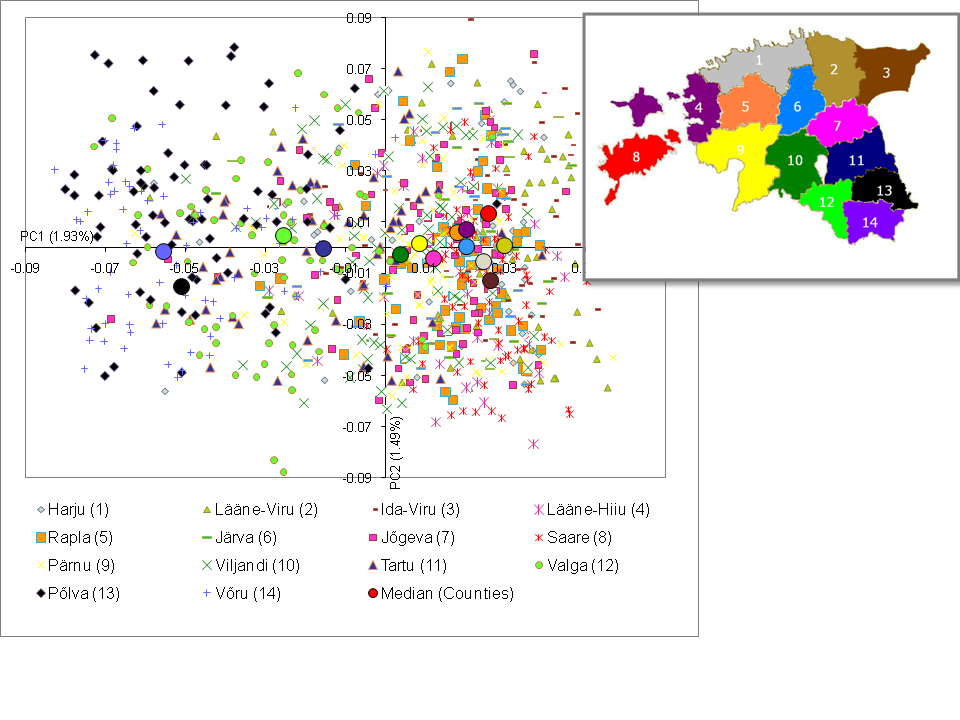

Supplement: Figure S1 — PC map of Estonian counties. Shown are the Estonian samples grouped by county. The great circles mark the median PC values for each county. Counties are colour-coded as shown in the inset. (0.25 MB TIF) [file pone.0005472.s006.tif]

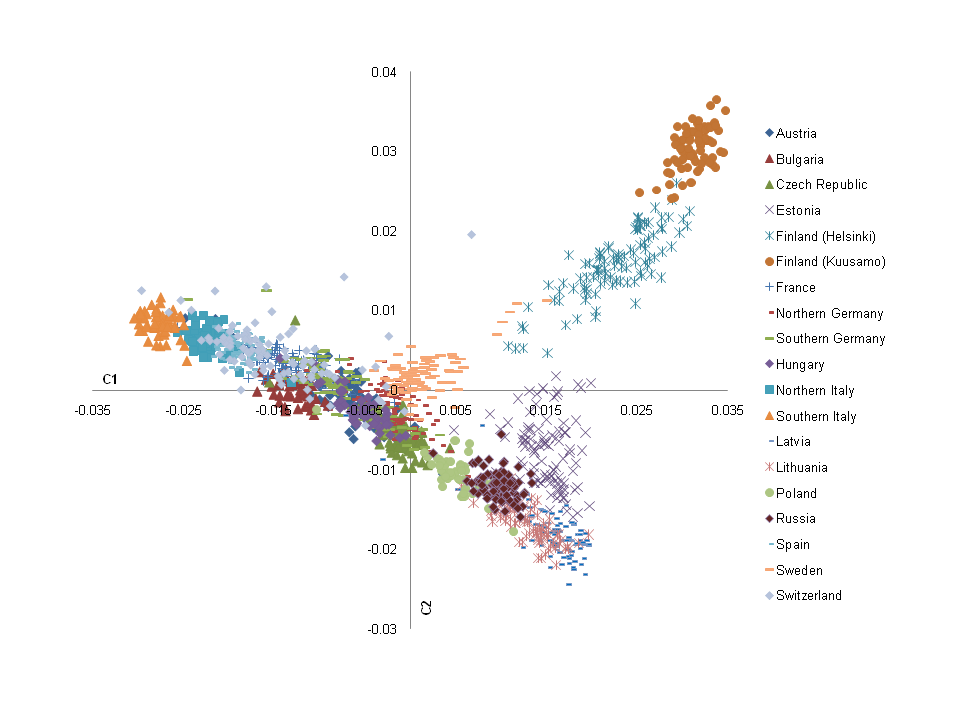

Supplement: Figure S2 — Multidimensional scaling plot of the studied European individuals. (0.14 MB TIF) [file pone.0005472.s007.tif]

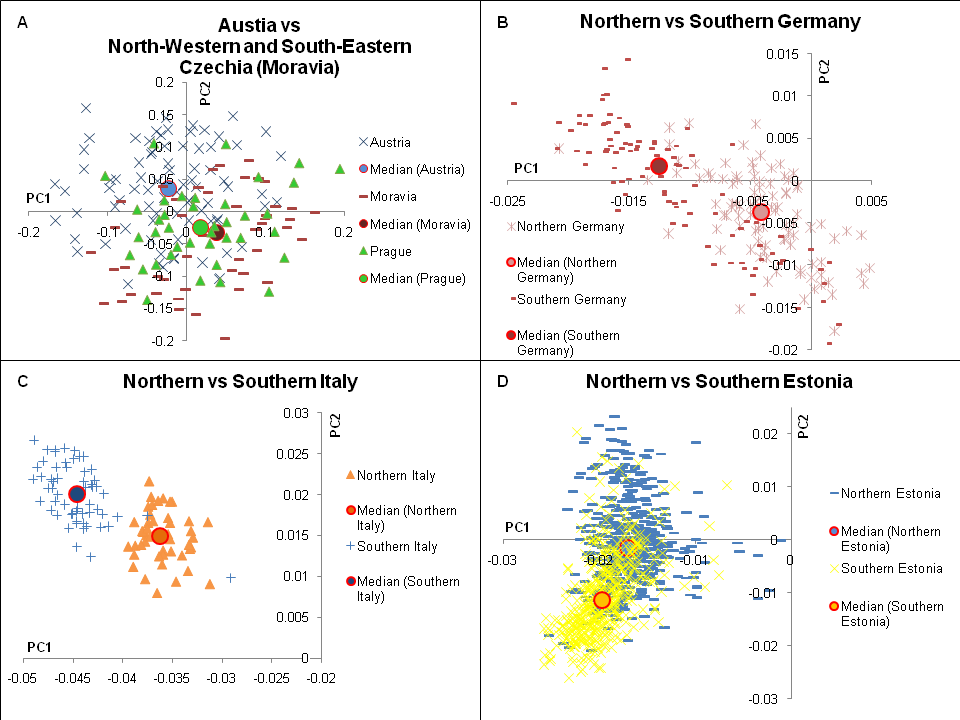

Supplement: Figure S3 — Population structure within studied populations. The scatter plots of the first two PCs show the level of stratification within A) Czech Republic - north-western part (Czech lands) and south-eastern part (Moravia), plus Austrian samples, B) Germany - northern and southern part, C) Italy - northern and southern part, and D) Estonia - northern and southern part. (0.15 MB TIF) [file pone.0005472.s008.tif]

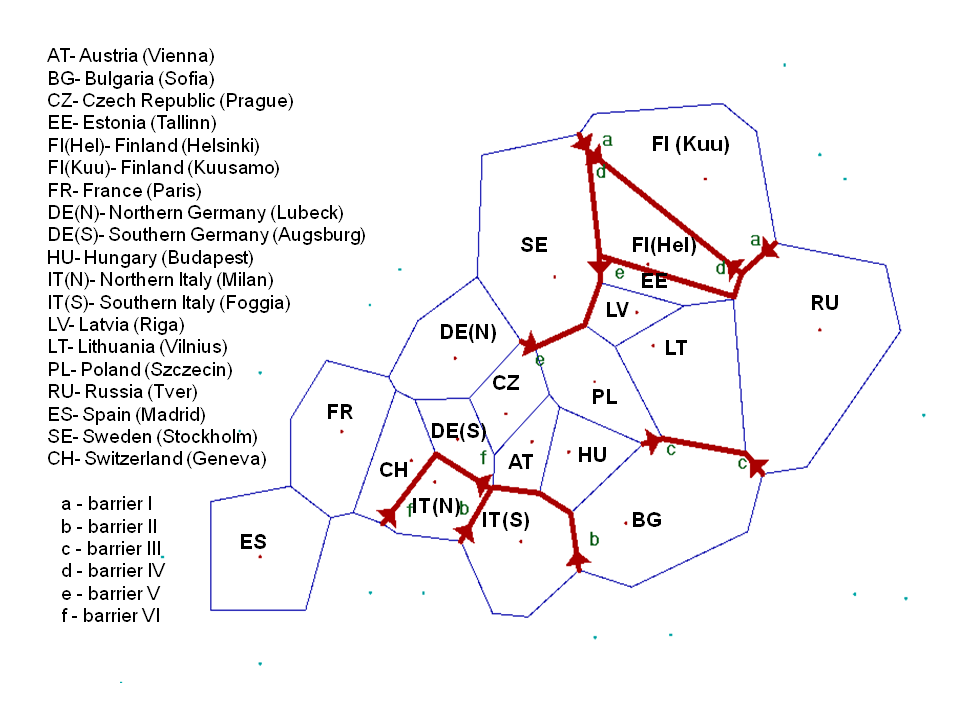

Supplement: Figure S4 — Barrier analysis of European gene pool. The analysis was based upon great-circle coordinates of the cities where individual population samples were recruited and pair-wise Fst. The name of the city is indicated in the brackets in the left panel. Lower case letters point the order of the found barriers. (0.15 MB TIF) [file pone.0005472.s009.tif]
